# First Report on Medical Treatment and Outcome of Burnt Cattle

**DOI:** 10.3390/vetsci10030187

**Published:** 2023-03-01

**Authors:** Marilena Bolcato, Mariana Roccaro, Arcangelo Gentile, Angelo Peli

**Affiliations:** 1 Department of Veterinary Medical Sciences, Alma Mater Studiorum University of Bologna, 40064 Bologna, Italy; 2 Department for Life Quality Studies, Alma Mater Studiorum University of Bologna, 47921 Rimini, Italy

**Keywords:** burn injuries, cattle, medical treatment, recovery, animal welfare

## Abstract

**Simple Summary:**

After a barn fire, the priority is to identify which animals are to be humanely killed for medical and/or ethical reasons. The decision to kill or treat cattle depends on the severity of the injuries but also the owner’s resources. On-farm treatment is possible, but it must be kept in mind that daily wound management and frequent monitoring need significant time and labor. Mild burns are unlikely to be fatal; however, it can take several days for the burns to appear in their full extent, leaving the prognosis uncertain. Clinical evidence regarding the specific management of bovine burn patients is lacking. This paper describes the clinical findings, treatment and outcome of two burnt Holstein heifers. Daily wound care required cleaning and the application of topical antibacterial agents. One heifer healed completely and was discharged after 7 months; the other one, after initial improvement, worsened due to the late onset of complications and was humanely euthanized. This proves that the treatment of burnt cattle is possible but challenging. More data on the success rate of medical therapy are urgently needed in order to confirm or refute whether this is an advisable option.

**Abstract:**

The management of livestock affected by fire often comes down to two options: euthanasia or slaughtering. However, the therapeutic approach can be attempted for high-value cattle. The aim of a primary assessment is to identify signs of smoke inhalation injuries, cardiovascular impairment and shock and to determine the severity and extent of burn injuries. Full-thickness burns covering 40% or more of the body are highly unfavorable prognostic factors and are usually fatal. Moreover, it can take several days for the burns to appear in their full extent, leaving the prognosis uncertain. In this case report, the clinical findings, treatment and outcome of two burnt Holstein heifers are described. Daily wound care required cleaning, the removal of eschars and the application of topical antibacterial agents for seven months in order to discharge one heifer. The topical use of honey with a solution of povidone–iodine proved to be affordable and successful, with no residue risks. The other heifer was more severely wounded, and despite the administration of fluid therapy, pain management, anti-oxidants and anti-microbials, after initial stabilization, the animal’s condition worsened, leading to euthanasia. This confirms that the treatment of burnt cattle is possible but challenging due to the late onset of multi-organ failure.

## 1. Introduction

Livestock farms are at risk of fire, because they contain fuels such as straw, hay or other dry foodstuffs, but also petrol, motor oil, etc. Barn fires often result from faulty or poor electrical wiring or equipment [1]. The effects of fires on animal health are attributable to the effect of heat and flames, resulting in more or less severe burns, but also to the effect of gases that may be released during combustion [2,3]. As soon as livestock can be approached, the first decision to be made is to identify which animals are to be ethically culled [4,5]. Euthanasia may be necessary for medical and/or ethical reasons, whereas slaughtering, whether on-farm or at the slaughterhouse, allows for partial compensation for the cost of the animal [6,7,8]. The latter is a common strategic option employed by Veterinary Services, and it complies with published government guidelines [9]. On the other hand, the therapeutic approach should not be ruled out *a priori*, although it is not without risk because, in case it fails, the recent administration of systemic analgesics or antibiotics might prevent the animal from being slaughtered, given the need to comply with the drugs’ withdrawal times. Moreover, since the therapeutic approach is only possible in mild cases, primary assessment is essential to determine the severity (superficial, partial or full thickness and subdermal) [10] and extent (total body surface area, TBSA) of burn injuries [2,11,12,13].

Unfortunately, there is currently no accurate method in veterinary medicine for the estimation of the affected TBSA. Given the diversity of conformations among veterinary patients, the translation of human estimation methods, such as the “rule of nines” or the “Lund–Browder chart” [14], onto animal patients will inevitably be inaccurate [11,15]. 

The treatment of burn wounds depends on the depth of the injury. Superficial burns may require the use of emollients and the application of antimicrobial substances such as topical antibiotics, povidone–iodine, silver compounds, silver sulfadiazine or chlorhexidine [12,14,15]. Recently, the use of honey has gained recognition because it is a safe natural substance, effective in inhibiting bacterial growth and containing a wide variety of active compounds that can promote wound healing [16,17]. Deep burns, instead, are characterized by the presence of necrotic eschar which must be selectively removed, with minimal blood loss, and then covered with topical antimicrobial substances until the wound is closed or a graft is applied [13,15,18].

During the clinical examination of burnt cattle, special attention should be paid to sensitive, painful or long-healing structures, such as eyes, hooves, teats and scrota [3,4,8]. Identifying signs of respiratory distress, smoke inhalation injuries, cardiovascular impairment, shock and any concurrent injuries is likewise critical [11,14,15]. In addition to that, one should always keep in mind that these medical classifications do not consider the emotional and welfare impact of being involved in a fire. However, it is acceptable to assume that fire-related injuries in livestock affect animal welfare, if for no other reason than the high level of pain caused by these injuries [19].

In small-animal veterinary practice, considerable attention is paid to resuscitation, ventilation and forced nutrition, and several medical and surgical options for wound treatment are described [11,15]. On the contrary, consensus guidelines and clinical evidence regarding the specific management of bovine burn patients are lacking. Nevertheless, the possibility that, as time progresses, multi-systemic, often unpredictable internal injuries become manifest, resulting in an inauspicious outcome of treatments, remains the greatest challenge in all species [11,15,20,21,22]. 

This article aims to describe the clinical findings, treatment, and outcome of two heifers involved in a barn fire.

## 2. Case History

A Holstein dairy farm consisting of three separate facilities, i.e., one for adult cows, one for heifers and one for calves, was involved in a fire. The fire originated from one of the individual cages in which the calves were housed due to a short circuit in the UV lamp system and quickly spread from one cage to another as these were placed side by side and next to the heifers’ facility. A total of 18 animals died; all 13 calves housed in single cages died during the fire, whilst 5 out of the 9 heifers died during the following week. None of the adult cows, housed further away from the young animal area, were affected by the fire. The surviving four 4-month-old heifers were examined on the farm one week after the fire. All animals underwent a complete physical examination; complete blood counts and blood chemistry profiles were also performed. 

## 3. Results

### 3.1. On-Farm Clinical Examination

**Figure 1:**
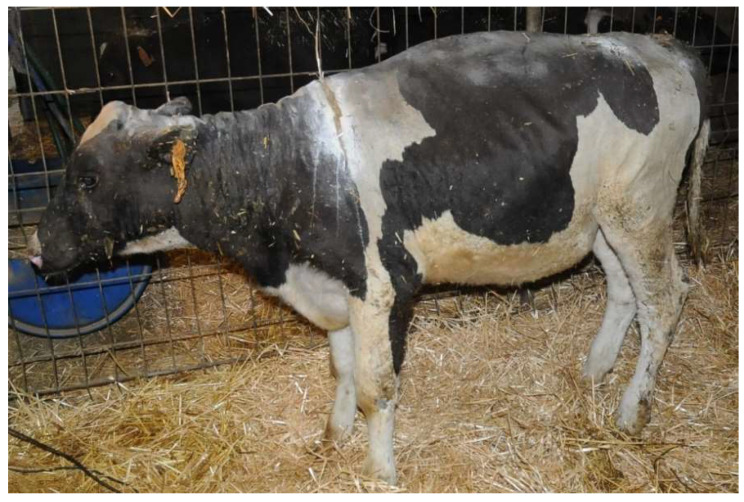
Heifer 1 at first clinical examination. Note the wide split in the skin at the neck level.

Heifer 1 presented depressed demeanor, reluctance to move and stiff gait. The head, the neck, all the dorsum and bilaterally the chest were hairless with dry and inelastic skin, covered by multiple and diffuse bubbles. At withers, there was a deep and complete split of the skin (Figure 1). The ears were short and curled, the eyelids had no eyelashes, and the protrusion of the third eyelid and paralysis of the eyelids were observed. Paralysis of the tail was also detected. The temperature was 37.5 °C, the pulse rate was 145 bpm and the breath rate was 40 breaths per minute.

**Figure 2:**
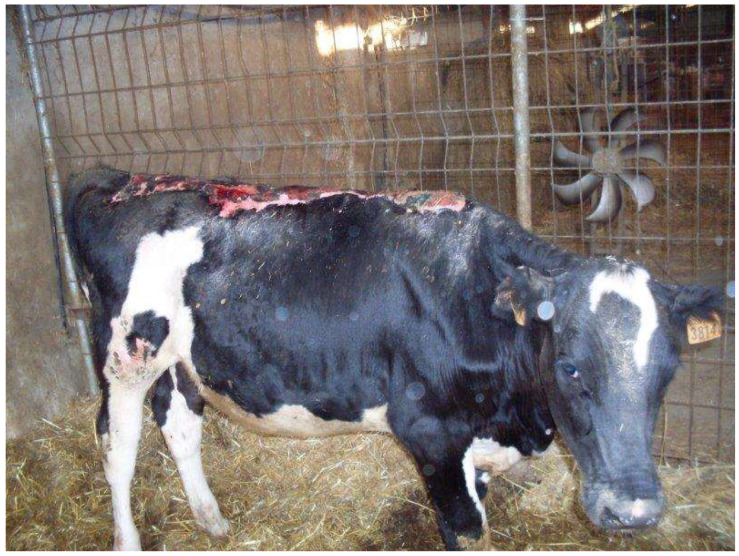
Heifer 2 at first clinical examination, which showed quadrupedal station and alert demeanor. Note the skin lesion on the back.

Heifer 2 presented normal demeanor, reluctance to move and stiff gait. The head, the neck and all the dorsum showed hairless black, hard, inelastic skin and a deep partial-thickness wounds (Figure 2). The temperature was 39.5 °C, the pulse rate was 140 bpm and the breath rate was 35 breaths per minute.

Heifer 3 and Heifer 4 showed mild clinical signs such as the singeing of hairs, normal demeanor and no respiratory signs; no foot burns were present, but reluctance to move and stiff gait were evident. They were therefore treated on site by the farm veterinarian, and the treatment and outcome are not reported in this paper.

Due to their poor health conditions and the owner’s request to try and save these animals, Heifer 1 and Heifer 2 were admitted to the teaching hospital of the Department of Veterinary Medical Sciences of the University of Bologna. Given the lack of literature regarding hospitalized burnt cattle, the treatment provided was reportedly guided by treatment recommendations for other burnt livestock species (i.e., horses, goats, sheep and pigs) in intensive care or hospital settings [20,21,22].

### 3.2. Recovery, Treatment and Outcome

#### 3.2.1. Heifer 1

At the day of admission, Heifer 1 presented full-thickness burns encompassing the epidermis and extending into the hypodermis, which involved approximately 30% of the body surface, mainly located on the back, caudally until the rump and ventrally until Vogël’s lower line. Namely the muzzle was burnt, the eyelashes absent and the eyelids immobile. The third eyelid was present and reactive, as was the eyeball, after testing the eye reflexes; the ears were deformed and crumpled, as were the ear tags. The tail was completely paralyzed. All over the body, the skin was hairless, covered with millet-seed-sized bubbles. To the touch, the skin was cold and leathery. On the back, at withers level, there was a deep transverse split in the skin, which at every (rare) movement of the animal revealed the underlying subcutaneous tissue and muscle bands, both of which were dry in appearance and smelt nauseating. The animal was in a reduced sensory state. Cranial and panniculus reflexes were present and physiological. The hooves showed no burns of the coronary band or heel bulb, and there were no signs of hoof detachment. However, the animal preferred to lie down, and when standing, it kept shifting weight away from the limbs. The mucous membranes were pink and the explorable lymph nodes normal. The temperature was 39 °C, the pulse rate was 140 bpm and the breathing was 40 acts per minute. Major organ functions were reduced but present. Blood biochemical examinations showed increased platelet count (1,198,000/mm^3^), neutrophilia (25,871/mm^3^), leukocytosis (33,740/mm^3^), increased lactatemia (3722 IU/L), creatine kinase (1931 U/L) and alkaline phosphatase (456 U/L). Moreover, the highest alteration to the coagulation profile was in the fibrinogen concentration (26.39 g/L). 

**Figure 3:**
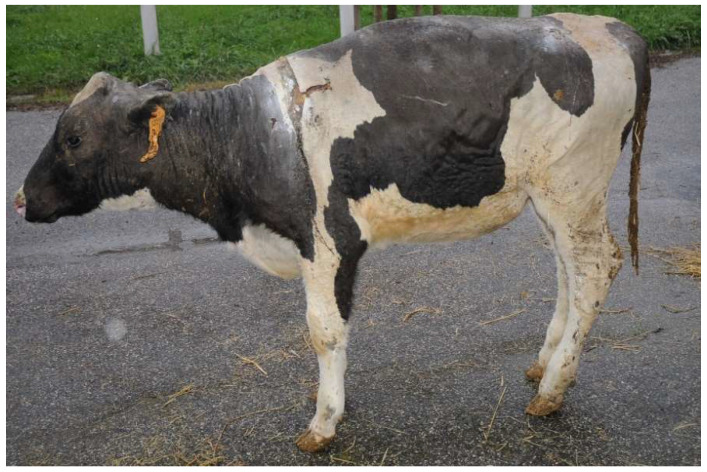
Heifer 1’s skin lesions three days after admission. Note the disjunction at the level of the fissure present on the neck and the thickening of the skin at the level of the ribs and abdomen.

The heifer was housed in a heated box, with deep straw bedding and with ad libitum access to good-quality hay and water. Fluid therapy consisting of 4 L of ringer lactate per day, antibiotic therapy (sodium/benzylpenicillin/procaine plus benzylpenicillin/dihydrostreptomycin 6,000,000 UI IM every 24 h), pain therapy (ketoprofen 3 mg/kg IM every 24 h) and vitamin and antioxidant support (selenium/tocopherol/cyanocobalamin 0.06 mg/Kg IM every 24 h) were administered during hospitalization. Skin lesions were treated BID and consisted of removal of the eschar, cleaning of the wound with diluted betadine and hydrogen peroxide as well as the application of topical antibiotics (gentamicin 0.1%) directly to the wound (Figure 3).

**Figure 4:**
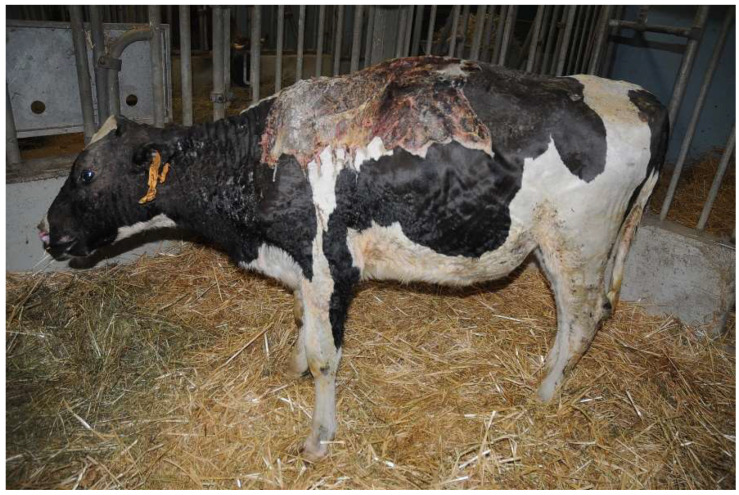
Heifer 1’s clinical appearance ten days after admission.

Clinical conditions were stable for two weeks (Figure 4); the heifer showed the ability to stand up and stay, regained tail movement, had increased appetite and had normal organic functions. Unfortunately, during the following five days of recovery, the heifer’s conditions worsened; it was not able to maintain the quadrupedal stance, its appetite decreased and recumbency became prolonged. Blood biochemical examinations showed progressive worsening of all parameters, but in particular, neutrophils (34,620/mm^3^), leukocytes (43,250/mm^3^), alkaline phosphatase (739 U/L, urea (26.70 mg/dL), glucose (106 mg/dL) and total cholesterol (33 mg/dL) concentration. Even potassium (3.7 mEq/L) and chlorine (89 mEq/L) showed severe abnormalities. Coagulation tests, such as the prothrombin time (52.7 s), activated partial thromboplastin time (61.1 s) and fibrinogen levels (4.83 g/dL), strongly suggested a condition of disseminated intravascular coagulation (DIC). Detailed results of the blood biochemical and coagulation profiles can be found in Tables S1 and S2. Therefore, due to the severe welfare impairment, the animal was humanely euthanized.

#### 3.2.2. Heifer 2

On the day of admission, Heifer 2 presented superficial and deep partial thickness burns on the 10% of the body surface, localized over the neck and the back. Here, the skin was hairless, hard and inelastic. Local burn injuries on the lateral side of the hind limbs were also present. The ventral part of the abdomen and udder and teats seemed unaffected. No hoof pain or separation was detected. The sensory state was alert, and the cranial reflexes were present and physiological, as well as the panniculus reflex; the mucous membranes were pink and the explorable lymph nodes normal. The heifer was able to stand up and stay, but kept shifting weight away from the limbs, and was reluctant to walk. The temperature was 39 °C, the pulse rate was 140 bpm and the breathing rate was 35 acts per minute. The blood biochemical examinations showed increased platelet count (1,035,000/mm^3^), neutrophilia (11,620/mm^3^), increased lactatemia (2598 IU/L) and alkaline phosphatase (523 U/L). Detailed results of the blood biochemical and coagulation profiles can be found in Tables S1 and S2.

The heifer was housed in a heated box with deep straw bedding and ad libitum access to good-quality hay and water. Fluid therapy consisting of 4 L of ringer lactate per day, antibiotic therapy (sodium/benzylpenicillin/procaine plus benzylpenicillin/dihydrostreptomycin 6,000,000 UI IM every 24 h), pain therapy (ketoprofen 3 mg/kg IM every 24 h) and vitamin and antioxidant support (selenium/tocopherol/cyanocobalamin 0.06 mg/Kg IM every 24 h) were administered for 10 days. Concurrently, skin lesions were treated BID, consisting of the removal of the eschar, cleaning of the wound with diluted betadine and hydrogen peroxide, as well as the application of topical antibiotics (gentamicin 0.1%) directly to the wound. 

**Figure 5:**
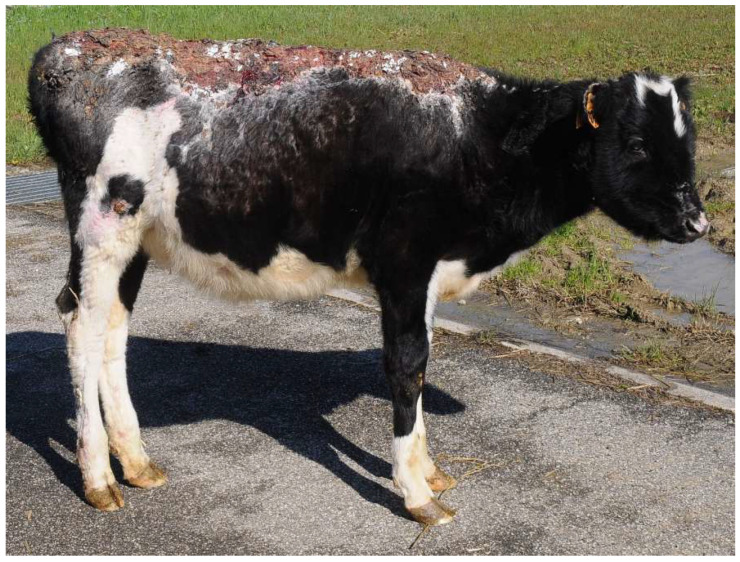
Heifer 2 three months after admission.

**Figure 6:**
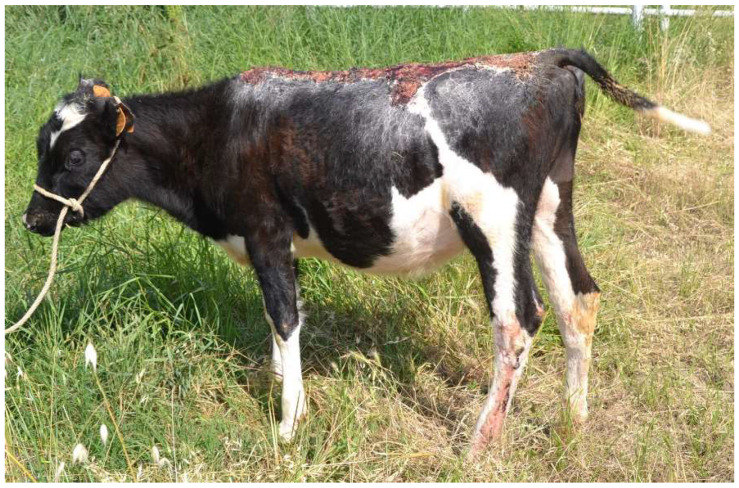
Heifer 2 five months after admission.

During hospitalization, gradual and progressive degradation and regeneration of the skin of the neck and back, in a ventro-dorsal direction, was observed (Figure 5 and Figure 6). Large areas of skin were removed each time, taking care to keep the wound margins clean and vital. Various medications were used on the wounds: a topical antibacterial was initially applied, and once the skin regeneration process had begun, skin regeneration stimulators containing hyaluronic acid were applied, but without satisfactory results. A honey plus betadine cream solution was therefore opted for. To ensure comfort and protection, the animal was also treated against ecto- and endoparasites (ivermectin 200 mcg/Kg IM).

After seven months of hospitalization, the animal was finally discharged. Afterwards, the owner reported that the heifer was inseminated and had a regular pregnancy and eutocic delivery. However, at the time of milking, two teats turned out to be completely obliterated, and the animal was therefore culled.

## 4. Discussion

Although there are several treatment possibilities available for burnt animals, the management of cattle affected by fire often comes down to two options: immediate euthanasia or slaughtering. However, the therapeutic approach can be attempted for high-value animals, provided that patients have mild injuries and no respiratory or cardiovascular damage. Cost must be taken into account, also considering that possible complications or the subsequent impairment of production performance could still lead to culling. The treatment protocol requires time and personnel, which are often insufficient in the on-farm scenario. On the other hand, hospitalization can be expensive, veterinary hospitals suitable for the hospitalization of large animals are not numerous and their capacity is usually limited.

In the immediate assessment, attention should be focused on the evaluation of the respiratory, cardiovascular and integumentary systems and the determination of the severity and extent of the burn injuries. Full-thickness burns covering 40% or more of the body are highly unfavorable prognostic factors and usually fatal [23]. Severe burns cause systemic damage that requires fluid therapy, analgesia, and daily wound management. 

Considering the therapeutic options, the medical protocols (antibiotic therapy, pain therapy, vitamin support and topical wound treatment) are not much different from the treatment of other common diseases, although the required time and labor are undeniably long and demanding. Therefore, the choice is always the result of a risk–benefit analysis, although this is made more complex by the uncertainty of the prognosis. This becomes even more challenging when dealing with burnt cattle. 

The cases presented in this study deviate from what is normally done since the most common decision is to cull burnt livestock. However, in this situation, the farmer was more interested in saving the animals with particular genetic value and to bear the costs of hospitalization despite the risk of treatment failure. Unfortunately, in this case, the outcome was unfavorable for both the animals.

In the case of Heifer 1, despite the extensive and moderate deep burns, considering the presence of organic functions, sensory status and the absence of signs of acute pain or hoof detachment, a therapeutic approach was attempted. Nevertheless, burns may take several days or weeks to become evident in their full extent. Burnt patients have a high risk of developing renal failure related to the release of myoglobin and other tissue catabolites linked to the necrosis of damaged tissues that are released into the blood stream. Moreover, due to traumatic stress, the metabolism of the burnt patient assumes a strongly catabolic pathway at a time when the animal’s appetite is at its lowest. Lastly, it must also be considered that the massive release of proinflammatory mediators has severe consequences on coagulation homeostasis. In our case, all these burn-associated syndromes developed and led to a fatal multi-organ syndrome, with nephrosis and myocardiosis.

Heifer 2, which showed moderate signs of burns on 10% of the body surface, did not develop post-burn syndromes, and the clinicians’ attention could focus on wound management. Full-thickness burns were left to heal by secondary intention (exposed technique) with the topical application of a honey plus betadine cream solution. Since scab formation may preclude wound evaluation and cause transient leukopenia, skin hypersensitivity and the development of bacterial resistance, the cleaning and disinfection of the area was carried out before each topical treatment, performed twice a day during the first post-burn period (3 months), then daily.

The most commonly used topical antibacterial for the treatment of burn wounds is silver sulfadiazine; other effective topical antimicrobials include chlorhexidine, povidone-iodine, and gentamicin sulfate ointment. Burn wounds heal slowly, and it may take many weeks for a wound to close by means of granulation, contraction and epithelialization. Burn wound healing by secondary intention occurs in three weeks if the wound is superficial; conversely, deep partial-thickness wounds require several months to heal, during which time, the bacterial contamination of the wound might develop. To overcome this, the topical use of honey with a solution of povidone–iodine guarantees an affordable and successful result, which does not pose residue risks. However, even when the clinical course has been favorable, it must never be forgotten that deeper and apparently invisible lesions may exist. This is what happened in this second case, where the diagnosis of teat obliteration/fusion could only be made at first lactation. It must be noted that, on clinical examination performed at the moment of admission, the heifer had no lesions in the udder region. It can be hypothesized that this condition might not necessarily have been a consequence of the fire but a congenital defect. Unfortunately, as this animal was a heifer, the condition only became evident at first lactation; however, the possibility to cull the animal after first delivery and have minimal economic payback was still satisfactory for the farmer.

## 5. Conclusions

When dealing with burnt livestock, it is advisable to be practical and effective according to the context and the available resources. Although hospitalization remains a hardly feasible option and the on-farm therapeutic approach is demanding in terms of time and labor, they can be considered for cattle with high value showing mild burns. Our case report describes a unique example of the medical treatment and hospitalization of burnt cattle, confirming that mild burn injuries extended to less than 40% of the body surface can be adequately treated. Instead, deep burns cause alterations that require aggressive treatment such as fluid therapy, medical therapy, frequent monitoring and daily wound management. However, the possible occurrence of complications such as hypothermia, infection, DIC, and multi-organ failure must be considered in the prognosis. 

This paper provides useful information to veterinary practitioners that might find themselves in the same situation, which is a possibility that will likely increase over time due to the challenges posed by climate change. These data could also be translated to the care of wild (endangered) species involved in wildfires. Further studies that explore different treatment options and add data on the success rate of medical therapy are urgently needed in order to confirm or refute whether this is an advisable strategy.

## Data Availability

All study data are contained within the article or supplementary material.

## Supplementary Materials

The following supporting information can be downloaded at: https://www.mdpi.com/article/10.3390/vetsci10030187/s1, Table S1: Blood biochemical parameters on the day of admission (T0) and after 15 days (T15) of Heifer 1 and Heifer 2; Table S2: Coagulation parameters on the day of admission (T0), after 15 days (T15) and on the day of euthanasia (T20) for Heifer 1 and after 30 days (T30) of hospitalization for Heifer 2.
