# Supplementary material for: First Report on Medical Treatment and Outcome of Burnt Cattle
Source: Vet Sci. 2023 Mar 1;10(3):187. doi: 10.3390/vetsci10030187 (PMC10058702; doi:10.3390/vetsci10030187)
Supplement: Supplementary file 1 [file vetsci-10-00187-s001.zip › vetsci-2187569-supplementary.pdf]

veterinary sciences

MDPI

Case Report

First Report on Medical Treatment and Outcome of Burnt Cattle

Marilena Bolcato [1,*], Mariana Roccaro [2], Arcangelo Gentile [1], Angelo Peli [2]

[1] Department of Veterinary Medical Sciences, Alma Mater Studiorum University of Bologna, Ozzano dell'Emilia 40064 - Bologna, Italy
[2] Department for Life Quality Studies, Alma Mater Studiorum University of Bologna, 47921 - Rimini, Italy
* Correspondence: marilena.bolcato2@unibo.it

Table S1. Blood-biochemical parameters at the day of admission (T0) and after 15 days (T15) of heifer 1 and heifer 2.

Parameter (measure unit)	Heifer 1		Heifer 2	
	T0	T15	T0	T15
Haemoglobin (gr%)	7.8	10.3	7.9	11
Haematocrit (%)	24.7	29.6	24.9	31.9
Erythrocytes (/mm³)	6850000	8440000	7310000	9470000
Platelets (/mm³)	1198000	855000	1035000	1059000
Leukocytes (/mm³)	33740	43250	16790	17630
Lymphocytes (/mm³)	5540	7020	3290	4230
Monocytes (/mm³)	870	600	1270	840
Neutrophils (/mm³)	25870	34620	11620	11850
Eosinophils (/mm³)	810	260	250	430
Basophils (/mm³)	430	640	180	200
Aspartate Aminotransferase (U/L)	292	226	103	77
Lactate Dehydrogenase (IU/L)	3722	3282	2598	2654
Creatine Kinase (U/L)	1931	789	396	1095
Alkaline Phosphatase (U/L)	456	739	523	456
Creatinine (mg/dL)	0.44	0.36	0.57	0.57
Urea (mg/dL)	11.80	26.70	22.33	17.66
Glucose (mg/dL)	62	106	61	89
Total Cholesterol (mg/dL)	58	33	82	59
Gamma-Glutamyl Transferase (U/L)	22.7	24	18.9	25.1
Total Protein (g/dL)	5.58	7.67	6.20	9.68
Albumin (g/dL)	2.12	1.63	2.47	2.33
Sodium (mEq/L)	139	135	136	137
Potassium (mEq/L)	5.1	3.7	4.5	4.3
Chlorine (mEq/L)	96	89	96	95
Magnesium (mg/dL)	1.87	1.12	1.94	1.51

veterinary sciences

MDPI

Table S2. Coagulation parameters at the day of admission (T0), after 15 days (T15), at the day of euthanasia (T20) for Heifer 1 and after 30 days (T30) of hospitalization for Heifer 2.

**Parameter (measure unit)**	**Heifer 1**			**Heifer 2**		
	**T0**	**T15**	**T20**	**T0**	**T15**	**T30**
Prothrombin Time (s)	19.2	32.2	52.7	23.3	22.0	21.0
Activated Partial Thromboplastin Time (s)	61.3	54.4	61.1	83.7	57.5	50.03
Fibrinogen (g/L)	26.39	20.62	4.83	9.97	6.09	5.98
D-Dimer (µg/mL)	4.71	2.70	3.30	3.79	1.21	0.11
Anti-thrombin III (%)	104	91	70	102	111	105
